# Eomes Expression Defines Group 1 Innate Lymphoid Cells During Metastasis in Human and Mouse

**DOI:** 10.3389/fimmu.2020.01190

**Published:** 2020-06-17

**Authors:** Riva Verma, Jun Zhi Er, Ren Wei Pu, Jameelah Sheik Mohamed, Ross A. Soo, Harish Mithiran Muthiah, John Kit Chung Tam, Jeak Ling Ding

**Affiliations:** ^1^Department of Biological Sciences, National University of Singapore, Singapore, Singapore; ^2^Division of Surgery, Yong Loo Lin School of Medicine, National University of Singapore, Singapore, Singapore; ^3^Department of Haematology-Oncology, National University Cancer Institute, Singapore, Singapore; ^4^Department of Cardiac, Thoracic and Vascular Surgery, Singapore, Singapore

**Keywords:** Eomesodermin, Group 1 ILCs, Innate Lymphoid Cells, metastasis, non-small cell lung cancer

## Abstract

Recent studies have attempted to uncover the role of Group 1 Innate lymphoid cells (ILCs) in multiple physiological contexts, including cancer. However, the definition and precise contribution of Group 1 ILCs (constituting ILC1 and NK subsets) to metastasis is unclear due to the lack of well-defined cell markers. Here, we first identified ILC1 and NK cells in NSCLC patient blood and differentiated them based on the expression of transcription factors, T-bet and Eomes. Interestingly, Eomes downregulation in the peripheral blood NK cells of NSCLC patients positively correlated with disease progression. Additionally, we noted higher Eomes expression in NK cells (T-bet^+^Eomes^hi^) compared to ILC1s (T-bet^+^Eomes^lo^). We asked whether the decrease in Eomes was associated with the conversion of NK cells into ILC1 using Eomes as a reliable marker to differentiate ILC1s from NK cells. Utilizing a murine model of experimental metastasis, we observed an association between increase in metastasis and Eomes downregulation in NKp46^+^NK1.1^+^ Group 1 ILCs, which was consistent to that of human NSCLC samples. Further confirmation of this trend was achieved by flow cytometry, which identified tissue-specific Eomes^lo^ ILC1-like and Eomes^hi^ NK-like subsets in the murine metastatic lung based on cell surface markers and adoptive transfer experiments. Next, functional characterization of these cell subsets showed reduced cytotoxicity and IFNγ production in Eomes^lo^ ILC1s compared to Eomes^hi^ cells, suggesting that lower Eomes levels are associated with poor cancer immunosurveillance by Group 1 ILCs. These findings provide novel insights into the regulation of Group 1 ILC subsets during metastasis, through the use of Eomes as a reliable marker to differentiate between NK and ILC1s.

## Introduction

Since the description of TRAIL^+^ NK cells in mouse liver in 2001 (1), extensive progress has been made in identifying the phenotype and function of recently discovered Innate Lymphoid Cells (ILCs) in health and disease (2–8). In both human and mouse, ILCs are the innate immune counterparts of T-cells, with ILC1, ILC2, and ILC3 sharing features with Th1, Th2, and Th17 subsets, respectively. Together with the previously identified and well-studied natural killer (NK) cells, ILC1s have been categorized as Group 1 ILCs. The NK cells represent the cytotoxic counterparts of ILCs, bearing similarity to CD8^+^ T cells. Based on parallels drawn from T cells, the Group 1 ILCs are known to depend on T-bet for their development, and produce IFNγ upon activation (9–11). While both murine and human ILC subsets are defined in a similar way, there is evidence suggesting differences in their precursor populations and pathways (12). The functions of Group 2 and Group 3 ILCs have been well-established through rigorous research efforts (13–20), however, that of Group 1 ILCs have remained somewhat unclear. This is, in part, due to lack of well-defined cell surface and intracellular markers to differentiate this heterogenous population into its subtypes—ILC1s and NK cells. As a result, most studies have referred to ILC1 subsets as tissue-resident NKs (tr-NK) or unconventional NKs, and this has led researchers to utilize markers common to both cell types, to define and study the roles of NK cells (21–23). Additionally, ILC1s and NK cells have been shown to exert different functions under different physiological conditions. Although, in the context of infectious diseases, the protective role of ILC1 has been uncovered (24), their contribution to tumor control and surveillance is controversial. While, it has been reported that loss of immune surveillance is associated with conversion of NK cells into ILC1s (25), another study has shown ILC1s to exhibit potent cytotoxicity against cancer cells (26). Furthermore, a high degree of plasticity amongst various ILC subsets (27, 28) makes it even more challenging to identify and examine the roles of different Group 1 ILC subsets in disease and pathology (25, 29, 30). Therefore, since it is now known that other subsets in addition to NK cells might exist, there is a need to revisit these studies and characterize the actual individual contribution of the Group 1 ILC subsets in order to reliably associate their specific functions to different diseases.

Early attempts to differentiate between NK cells and ILC1s utilized CD49a and CD49b (DX5) as two mutually exclusive markers (31). Classical NK cells in the bloodstream as well as in thymus, liver, skin, and uterus predominantly express CD49a^−^CD49b^+^ phenotype. On the other hand, ILC1s in the liver, skin and uterus are CD49a^+^CD49b^−^. However, a recent study has identified an intermediate ILC1 population which expresses CD49a^+^CD49b^+^ phenotype in the tumor microenvironment (25). Further, it is also noteworthy that the upregulation of CD49a and downregulation of CD49b occur under inflammation conditions in activated NK cells, thereby making these markers non-specific to the subsets (32). Therefore, alternative strategies have been tested to distinctly define the two subsets. Developmental dependence of NK cells on transcription factors such as NFIL3 (33) and that of liver ILC1s on Hobit (34) has been explored in attempts to study the individual function of these cells. However, the results from these trials have been rather unsatisfactory due to development of certain NK cells even in *Nfil3*^−/−^ and ILC1s in *hobit*^−/−^ mice in the presence of inflammatory stimuli. Likewise, a recent study identified CD200r1 as an ILC1 specific marker in the liver. However, its expression on ILC1s in other organs is unknown (35). For the purpose of this study, we defined Group 1 ILCs based on expression of T-bet and Eomesodermin (Eomes) in mouse and human. T-bet is a T-box transcription factor needed for the development of Group 1 ILC subsets while Eomes is needed for NK cell development, specifically (36). While T-bet is expressed on both ILC1s and NK cells (37), Eomes is seemingly expressed only on murine NK cells (9), thus making it a more reliable marker to differentiate ILC1s from NK cells (11, 38, 39).

Emerging studies have queried the involvement of novel Group 1 ILC subsets in disease and pathology (40–42), but little is known about their phenotype and function in cancer. Recently, Dadi et al. found an immuno-surveillance role for murine ILC1-like cells in genetic models of murine mammary carcinoma (26). On the other hand, an immune-suppressive role of human CD56^+^CD3^−^ Group 1 ILCs in Tumor Infiltrating Lymphocyte (TIL) culture has been reported (43). In the context of metastasis, while the role of NK cells is well-studied (44), that of recently identified ILC1 subsets is unknown (45–48). Here, we aimed to study Group 1 ILC subsets involved in metastasis by analyzing the profile of Group 1 ILCs in blood samples of NSCLC patients. We identified distinct ILC1 (Eomes^lo^) and NK cells (Eomes^hi^) in patient blood and observed Eomes downregulation in Group 1 ILCs (NK cells in particular), with the advancement of post-metastatic NSCLC. Similarly, using a mouse model of metastatic melanoma, we identified T-bet^+^Eomes^lo^ and T-bet^+^Eomes^hi^ subsets within NKp46^+^NK1.1^+^Group 1 ILCs. Subsequent *ex vivo* analysis of the Group 1 ILC subsets showed increased cytotoxicity with increased Eomes expression. Based on our findings, we propose that the Eomes levels regulate the response of Group 1 ILCs to metastasis. Furthermore, the weakening of Group 1 ILC anti-tumor response was associated with Eomes downregulation, which could contribute to worse clinical outcomes in cancer metastasis.

## Materials and Methods

### Patient Samples

All patient samples used in this study were collected from the National University Hospital (NUH), Singapore, approved under DSRB number 2016/00698 and were taken after patient written informed consent at least 24 h before the surgery or on the day of the consultation. Five milliliter of peripheral blood was collected from NSCLC patients before the treatment was started. Stages I and II samples were collected from patients undergoing surgical resection of lung mass while Stages III and IV were collected from patients consulting with National University Cancer Institute (NCIS) at NUH. De-identified patient information is provided in Table S1. Blood specimens were diluted 1X with HBSS and layered onto ficoll-paque media (GE Healthcare) and centrifuged at 400 g for 40 min at 20°C without brake and acceleration, after which the PBMC ring was collected into a fresh tube. The cells were then washed twice, counted and shifted to ice for immunostaining and flow cytometry.

### Flow Cytometry of Human PBMCs

Cells were resuspended in 1 ml PBS and spun down at 500 g for 5 min at 4°C. The cells were then stained for 30 min with a live-dead stain, Fixable Viability Dye (FVD)-506 at 1:1000 dilution in 100 μl PBS. Then, the cells were washed and stained for cell-surface markers. In order to improve the antibody binding, a blocking antibody (Biolegend) was used at 1:200 dilution. A lineage panel consisting of the following antibodies was included to allow for clear identification of ILCs—FITC-conjugated anti-CD3 (OKT3), anti-CD19 (H1B19), anti-CD11b (M170), anti- CD11c (3.9). To this mix, the following antibodies from Biolegend were added at 1:50 dilution: APC-Cy7-conjugated anti-CD45(2D1), PerCP-conjugated anti-CD56 (CMSSB), PE-Cy7-conjugated anti-CRTH2 (BM16), PacBlue-conjugated anti-CD117 (104D2) and Qdot-605-conjugated anti-CD127 (A019D5). Cells were incubated with the antibodies for 30 min on ice. This was followed by fixation permeabilization for detection of intranuclear T-bet and Eomes markers. For this, eBioscience Foxp3 transcription factor staining kit was used (#005523), following which the cells were stained with PE-conjugated anti-T-bet (4B10) and APC-conjugated anti-Eomes antibody (WD1928) at room temperature. Intranuclear staining with anti T-bet and Eomes antibodies was carried out 1 h before running the samples on flow cytometer. The cells were resuspended in 500 μl 2% FBS in PBS and centrifuged at 8,000 g to remove the supernatant. To the pellet, 400 μl of PBS was added before the suspension was filtered through 70 μm filter and run on flow cytometer. Fixed samples, prior to intracellular staining were stored overnight at 4°C. Samples were run on BD LSR Fortessa flow cytometer and analyzed using Flowjo V10. Fluorescence compensation data were acquired using single stained compensation beads (Thermofisher Scientific) and applied to the samples. For gating of positive and negative populations, Fluorescence Minus One (FMO) controls were used. For additional clarity, internal staining controls were used, wherever mentioned. For data presentation and statistical analysis, graphs were plotted using GraphPad Prism 5.01.

### Mice Models and Cell Lines

The experiments and breeding of mice were performed under Institutional Animal Care and Use Committee (IACUC approved protocols: R17-0209 and BR-1142, respectively). All the mice used in this study were housed at Comparative Medicine at MD1, National University of Singapore. Throughout this study, C57BL/6J female wild type (WT) mice between 6 and 8 weeks of age were used. T-bet KO and CD45.1 congenic mice were purchased from Jacksons lab while Eomes-GFP reporter mice were a kind gift from Dr. Thierry Walzer, Centre International de Recherche en Infectiologie, Inserm, Lyon, France. B16F10 melanoma cells were purchased from ATCC and were maintained in DMEM containing 10% FBS. Mouse melanoma B16F10 cells were tested to be mycoplasma-free. To set up a model of pulmonary metastasis in B6 mice, 0.2 million B16F10 cells in PBS were administered intravenously through the tail vein. Subsequently, the mice were euthanized, and lung and spleen tissues were harvested at different time points. The cells were then isolated and characterized as discussed below.

### Cell Isolation

White blood cells were isolated from mouse lungs, spleen, and liver using enzymatic and mechanical dissociation. After slicing the spleen into small fragments of ~2 mm, in PBS, a 10 ml syringe and plunger was used to release the cells further. For lung, tissue chunks were first incubated in 0.5 mg/ml Collagenase D and 20 U/ml DNase (Merck) for 20 min, followed by mechanical dissociation using Miltenyi tissue dissociator. Liver was perfused with 1 mM EDTA in PBS, isolated, and mechanically homogenized by using Miltenyi tissue dissociator. Dissociated tissue samples were then filtered through a 70 μm nylon filter (Miltenyi Biotec). For lungs and liver, the cells were resuspended in 40% Percoll PLUS density gradient medium (GE Healthcare) and overlaid on 70% Percoll Plus medium and centrifuged at 500 g for 30 min at 20°C The interphase containing lymphocytes was collected, washed and subjected to lysis of red blood cells using ACK lysis buffer, together with splenic cells. The isolated cells were then stained as described below.

### Flow Cytometry and Sorting of Group 1 ILCs

For staining of mouse samples, the following antibodies were used: From eBioscience: PacBlue-conjugated anti-CD45.1 (104), PE-Cy7-conjugated anti-NKp46 (29A1.4), APC/APC-Cy7-conjugated anti-NK1.1 (PK136), APC-conjugated anti-CD49b (DX5), PerCP-cy5.5-conjugated anti-CD11b (M1/70), AF488-conjugated anti-CD27 (LG.7F9), APC-conjugated anti-CD62L (MEL-14), FITC-conjugated anti-CD44 (IM7), PerCP-cy5.5-conjugated anti-Ki67 (So1A15), AF488-conjugated anti-IFNγ (XMG1.2), PerCP-cy5.5-conjugated anti-TNFα (MP6-XT22), PE conjugated anti-T-bet (4B10), PE-TexasRed-conjugated anti-Eomes (Dan11mag). PerCP-cy5.5-conjugated anti-CD49a (Ha31.8) was from BD Biosciences. A cocktail of biotin-tagged antibodies (eBioscience) containing anti-mouse CD3, CD19, CD5, γδTCR, TER119, Gr-1, F4/80 was used to separate NK cells from other immune cells. Fixable Viability Dye (FVD)-506 (65-0866, eBioscience) was used to separate live from dead cells, and cells were fixed using Foxp3 Fix/perm kit (88-8824-00, eBioscience). Flow cytometry of the cells was performed with BD LSR Fortessa and data were analyzed using FlowJo V10. In order to sort ILC1s/NKs, spleen from multiple Eomes-GFP reporter mice were pooled and processed to isolate single cells. For depletion of lineage cells, streptavidin beads were used to remove lineage-positive cells stained with biotin antibodies (StemCell™ Technologies, Catalog #19860). The cells were stained with PacBlue-conjugated anti-CD45.1 (104), PE-Cy7-conjugated anti-NKp46 (29A1.4), APC-conjugated anti-NK1.1 (PK136) and Eomes was detected using GFP expression. After staining, cells were sorted on BD FACS Aria and Eomes^lo^ and Eomes^hi^ Group 1 ILC fractions were collected in complete RPMI. MFIs (mean fluorescence intensity) of pre- and post-sorted cells were compared and a 99% pure population of Eomes^hi^ cells was isolated.

### Adoptive Transfer

2 × 10^5^ Eomes^hi^ cells were adoptively transferred into CD45.1 mice at day 4 post-B16F10 cancer cell injection. The lungs were then harvested and analyzed at day 10 using flow cytometry, and donor cells in the recipient CD45.1 mouse were detected as CD45.2^+^CD45.1^−^.

## Quantitative Reverse Transcription Polymerase Chain Reaction (qRT-PCR)

Flash frozen lung lobes from mice were thawed on ice and 500 μl Trizol was added to carry out RNA extraction. The lung lobe was finely excised and homogenized using a tissue dissociator (GentleMACS c tube, Miltenyi). Chloroform-isopropanol extraction was used to precipitate the RNA followed by another round of extraction with Trizol to achieve higher purity. The RNA pellet obtained thereafter was re-suspended in water and reverse-transcribed using superscript cDNA kit (ThermoFisher). The cDNA obtained was diluted 5 times and qRT-PCR using Promega master-mix was performed. All measurements were relative to reference gene, *Rpl27*, which was used as an internal loading control. The PCR primers for *Melan-A* were: F- 5′ GAGAAATCCCATCAGCCCGT 3′ and R- 5′ AGCGTTCTCAGGAGTTTCCC 3′, and for *Pmel* were: F- 5′ GCCACATGGTAGCACTCACT 3′ and R- 5′ AACAAAAGCCCTCCCGCAAG 3′.

### *Ex vivo* Stimulation and Intracellular Staining

IFNγ and TNFα production by murine Group 1 ILCs was measured through intracellular cytokine staining after *ex vivo* stimulation with 25 ng/ml Phorbol Myristate Acetate (PMA, Sigma) and 500 ng/ml ionomycin (Thermofisher Scientific) in complete RMPI at 37°C for 5 h. Secretion of the cytokine was blocked by the addition of GolgiPlug^TM^ (Beckton-Dickinson) to the media. Cells were then fixed using Foxp3 Fix/perm kit (88-8824-00, eBioscience) for 30 min on ice, and stained with anti-IFNγ AF488 and anti-TNFα antibody.

### Co-culture of Group 1 ILCs With Cancer Cells

For co-culture of mouse Group 1 ILC fractions with B16F10 cells, flow sorted Eomes^hi^ and Eomes^lo^ Group 1 ILC subsets were resuspended in RPMI and co-cultured with B16F10 cells in a 4:1 effector to target ratio. For measurement of Eomes MFI, cells cultured without B16F10 cells were used as controls and cytotoxicity was normalized against spontaneous cell death in “B16F10 only” wells. Cells were harvested onto ice at different time points and cytotoxicity was detected through 7AAD staining. The Eomes levels were measured through GFP expression. The cytotoxicity was determined by calculating [% dead cells / (% dead cells + % live cells)].

### Cytokine Administration and *in vivo* NK Cell Expansion

Intranasal administration of cytokines was carried out to evaluate the response of Group 1 ILC subsets to *in situ* stimulation. For this, the experimental mice were given 50 μl of 0.5 μg IL-12 and 1.0 μg IL-18 in 1x PBS while the control group received 50 μl of 1x PBS only. The mice were anesthetized in the gas chamber using isofluorane. This was done on days 1, 3, and 5, followed by harvesting and isolation of cells on day 7.

### Statistical Tests

Prism (GraphPad) was used for statistical analysis. Individual statistical tests used are described in the corresponding figure legend. *p*-values are shown in figures or included in the figure legend. For all animal studies, points represent biological replicates, for co-culture analysis technical replicates were used and representative experiments are shown. For NSCLC patient sample analysis, each point represents unique patient data. Bar position represents the mean, and error bars represent ± s.e.m.

## Results

### Eomes Downregulation in Circulating NK Cells Accompanied NSCLC Progression

**Figure 1:**
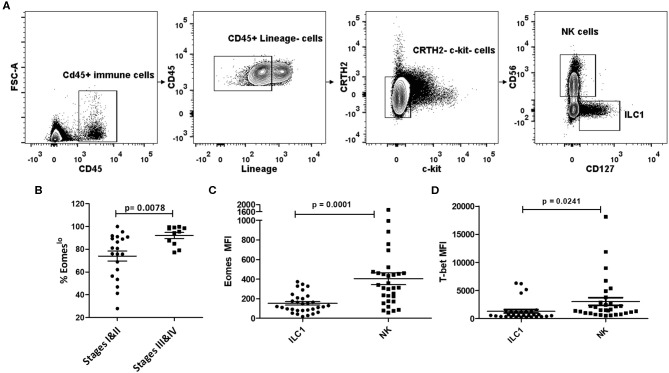
Human Group 1 ILCs expressed Eomes differentially during NSCLC progression. **(A)** Gating for identification of Group 1 ILC subsets in peripheral blood of NSCLC patients. Group 1 ILCs were gated as CD45^+^ Lineage (CD3, CD19, CD11b, CD11c)^−^c-Kit^−^CRTH2^−^; ILC1s were further gated as CD127^+^CD56^−^ and NK cells as CD127^−^CD56^+^ **(B)** Percentage of Eomes^lo^ subset gated over CD45^+^ Lineage (CD3, CD19, CD11b, CD11c)^−^c-Kit^−^ CRTH2^−^T-bet^+^ cells at early stage (Stage I and II) vs. late stage (III and IV). Cancer progression is accompanied with increase in frequency of cells expressing lower Eomes levels **(C)** Quantification of Eomes expression in Group 1 ILC subsets, viz, ILC1, and NK cells. Peripheral blood NK cells showed higher Eomes levels compared to circulating ILC1s **(D)** Quantification of T-bet expression in Group 1 ILC subsets, viz, ILC1, and NK cells. Peripheral blood NK cells showed higher T-bet levels compared to circulating ILC1s. MFI is Mean Fluorescence Intensity, *n* = 16 for stage I, *n* = 4 for stage II, *n* = 4 for stage III, *n* = 7 for stage IV. Data are presented as mean ± s.e.m.; significance was tested using unpaired two tailed students' *t*-test.

To investigate the role of Group 1 ILCs in cancer, we analyzed peripheral blood samples of NSCLC patients across various stages of cancer prior to treatment initiation. Group 1 ILCs in the blood were broadly classified as CD45^+^Lin^−^c-kit^−^CRTH2^−^ live cells and were subdivided into ILC1s and NK cells. ILC1s were defined as CD127^+^CD56^−^ and NK cells as CD127^−^CD56^+^, as described previously (49) (Figure 1A). Since the expression of T-bet and Eomesodermin (Eomes) on human Group 1 ILCs is not well-defined (39, 50, 51), we analyzed the profile of these transcription factors in circulating ILC1s and NKs in NSCLC. Interestingly, we noticed a decrease in the expression levels of Eomes post-metastasis (Stages III and IV) compared to early stage (Stages I and II), while T-bet levels did not change significantly (Figures S1A,B). We also noted a concomitant increase in the frequency of Eomes^lo^ cells (among the T-bet^+^ Group 1 ILCs) with NSCLC advancement (Figure 1B). This suggested downregulation of Eomes in Group 1 ILCs, particularly NK cells, during metastasis (Figures S1C,D). We also investigated Eomes levels in individual ILC1 and NK cell subsets and observed significantly elevated Eomes expression in NK cells, compared to ILC1s (Figure 1C). While T-bet was highly expressed in both NK cells and ILC1s, there was a notably higher expression in NK cells compared to ILC1s (Figure 1D). Based on this, we propose that circulating ILC1s exhibit T-bet^+^Eomes^lo^ profile while NK cells express T-bet^+^Eomes^hi^ phenotype during NSCLC progression (Figure S1E). Interestingly, we did not observe any significant difference in the ratio of NK cells to ILC1s with cancer advancement (Figure S1F).

### Murine Eomes^lo^ Group 1 ILCs Accumulated in the Lung and Spleen During Metastatic Progression

**Figure 2:**
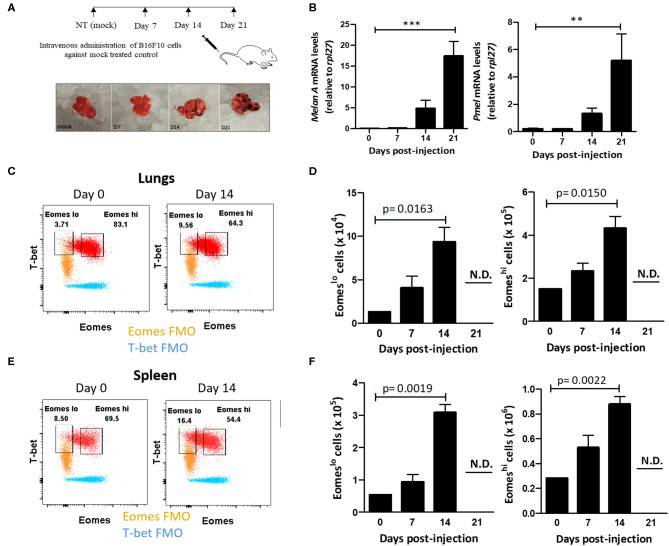
Eomes^lo^ Group 1 ILCs accumulate in the lung during B16F10 metastatic progression. **(A)** Schematic for setting up B16F10 murine metastatic melanoma study model in B6 mice. Mice were sacrificed and lungs were harvested at various time points. **(B)** Quantification of tumour burden in the lungs using melanocyte-specific genes *Melan-A* and *Pmel* relative to *Rpl27* housekeeping gene. **(C)** Identification and representative flow plot of pulmonary Eomes^lo^ and Eomes^hi^ cells in the lung at day 0 and day 14. **(D)** Quantification of Eomes^lo^ and Eomes^hi^ cell numbers with increase in metastatic burden in the lungs. Both these cell subsets were not detectable (N.D.) at day 21. **(E)** Identification and representative flow plot of splenic Eomes^lo^ and Eomes^hi^ cells at day 0 and day 14. **(F)** Increase in splenic Eomes^lo^ and Eomes^hi^ numbers with increase in metastasis. Both these cell subsets were not detectable (N.D.) at day 21. Cell populations were gated over NKp46^+^NK1.1^+^CD45.2^+^ live Group 1 ILCs for **(C–F)**. For **(C,E)**, FMO controls are overlaid. MFI is Mean Fluorescence Intensity, *n* = 4 for each group. Data are representative of three independent repeats; Data are presented as mean ± s.e.m.; statistical significance was tested using one-way ANOVA **(B)** two-tailed students' *t*-test **(D,F)**. **p < 0.005 and **p < 0.0005.

In light of these findings, we questioned whether Eomes downregulation in Group 1 ILCs during tumor progression was associated with acquisition of ILC1-like phenotype in NK cells. Furthermore, using a model of murine metastasis, we asked whether Eomes could also be used as a reliable marker to differentiate ILC1s from NK cells in the tumor microenvironment. After observing the loss of expression of Eomes in NK cells and increase in Eomes^lo^ ILC1s in human peripheral blood with cancer progression, we queried the profile of murine Group 1 ILCs during metastasis using a mouse model of B16F10 metastatic melanoma. To this end, we injected B16F10 cancer cells into the tail vein and monitored tumor development over 21 days (Figure 2A). The tumor burden in the lungs was quantified using qRT-PCR, based on *Melan-A* and *Pmel*, which are two melanocyte-specific markers with zero basal expression in mock-treated lungs (52). These genes were expressed at higher levels over time, indicating increased tumor burden in the lungs (Figure 2B). Next, we investigated the profile of pulmonary Group 1 ILCs during metastatic establishment and colonization. For this, we used the NK cell surface markers, NKp46 and NK1.1, to define Group 1 ILCs (9). Since Group 1 ILCs consist of ILC1s and NK cells which share similar phenotypic profile, we used Eomes as a marker to differentiate ILC1s and NK cells. Like with human samples, we probed for Group 1 ILC subsets in mice tissues based on Eomes and T-bet levels, revealing two different Group 1 ILC subsets: T-bet^+^Eomes^lo^ and T-bet^+^Eomes^hi^ cells in the lung and spleen (Figures 2C,E). Since ILC1s are generally Eomes^−^ but NK cells in most organs express Eomes (38), and our findings in human patient samples show that ILC1 expressed lower levels of Eomes compared to NK cells, we hypothesized that the pulmonary and splenic Eomes^lo^ and Eomes^hi^ subsets represent ILC1 and NK cells, respectively. Quantification of pulmonary and splenic Eomes^lo^ and Eomes^hi^ NK cells showed accumulation of these subsets with increase in tumor progression (Figures 2D,F). Furthermore, analysis of the ratio of Eomes^hi^ to Eomes^lo^ cells at different time points showed decrease in Eomes^hi^ cells with increase in metastatic burden, suggesting increase of the number of Eomes^lo^ cells in the lungs (Figure S2A). However, due to reduced infiltration of CD45.2^+^ immune cells into the lungs, we did not detect any Group 1 ILCs at day 21, coinciding with massive tumor burden at that time point (Figure S2B).

### Eomes^lo^ Group 1 ILC Subset Is Not Derived From Eomes^hi^ Cells in the Tumor Microenvironment

**Figure 3:**
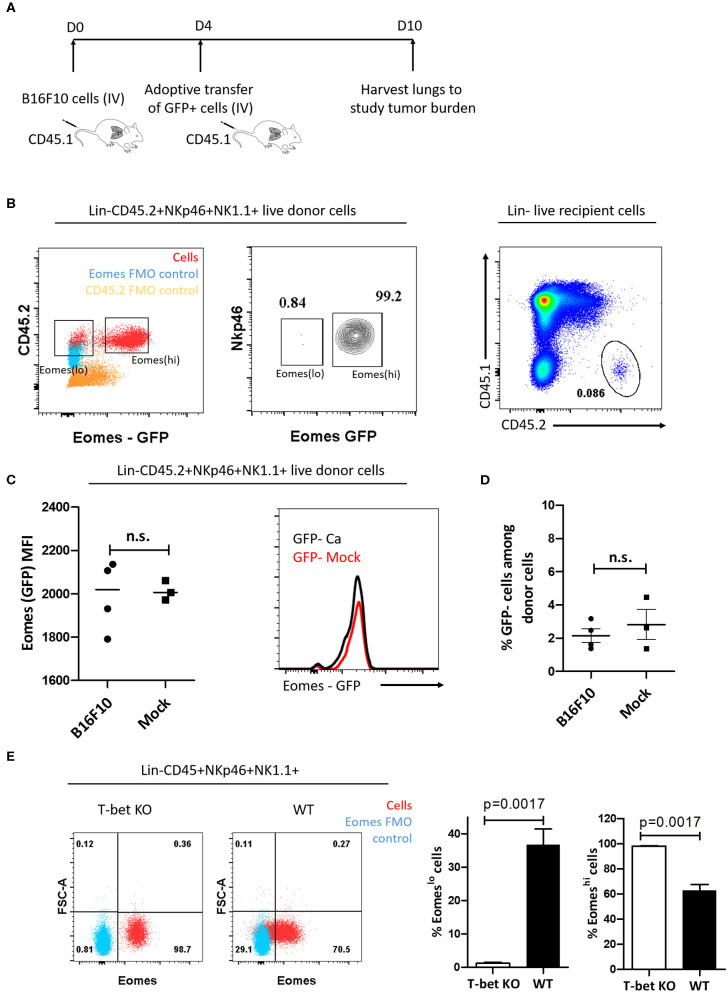
Eomes^lo^ and Eomes^hi^ subsets correspond to different cell lineages. **(A)** Scheme of adoptive transfer of Eomes^hi^ cells into CD45.1 mice bearing cancer (relative to mock). After injection of B16F10 cells (day 0), Eomes^hi^ cells were harvested from donor Eomes-GFP mice and adoptively transferred into recipient mice at day 4. The mice were then sacrificed at day 10 and lungs were harvested and analyzed. **(B)** Eomes^lo^ and Eomes^hi^ cells in Eomes-GFP mice (Red) overlaid with Eomes FMO (blue) (left panel). Two distinct populations can be seen upon running live cells under flow cytometer; Sorting efficiency ~99% middle panel); Donor cells after transfer of CD45.2^+^ Eomes^hi^-GFP^+^ cells into CD45.1 mice (right panel). **(C)** Eomes (MFI) after transfer of donor derived Eomes^hi^ GFP^+^ cells in cancer-bearing (B16F10) and cancer-lacking (mock) hosts at day 10. No significant difference in Eomes MFI was observed between cancer and mock mice. **(D)** Frequency of Eomes^lo^ GFP^−^ cells amongst donor NK cells at day 10 post-injection of B16F10 cells **(E)** Flow plots and graphs show near absence of Eomes^lo^ cells in T-bet KO mice (~0.8%) compared to WT mice (~29.1%), indicating that T-bet is needed for development of Eomes^lo^ cell development (Red—NKp46^+^NK1.1^+^ cells; Blue—Eomes FMO). MFI is Mean Fluorescence Intensity, *n* = 3–4 for each group. Results are representative of three independent repeats; data are presented as mean ± s.e.m.; Significance was tested using two-tailed students' *t*-test.

Cancer cells are known to polarize the tumor milieu in order to dampen the effector function of various immune cells (53). Since we identified increase in the frequency of T-bet^+^Eomes^lo^ population in the lung and spleen, we questioned whether the increase in Eomes^lo^ subset is due to Eomes downregulation in the tumor microenvironment, viz, could they have arisen from Eomes^hi^ cells? To this end, we first adoptively transferred FACS-sorted Eomes^hi^ cells isolated from the spleen of Eomes GFP reporter mice (CD45.2) into congenic CD45.1 mice at day 4 post-injection of B16F10 cells or PBS (mock). Since we observed an increase in Eomes^lo^ cells as early as day 7 after injection of B16F10 cells, we followed a similar timeline for adoptive transfer experiment. At day 10 after B16F10 injection, we analyzed the cell frequency and population (Figures 3A,B). However, we did not observe any decrease in Eomes expression (based on GFP MFI) in the adoptively transferred cells isolated from tumor-bearing recipient mice at day 10 compared to mock naïve recipient mice (Figure 3C). Likewise, we did not observe any increase in the frequency of Eomes^lo^ cell population upon the transfer of Eomes^hi^ cells into tumor-bearing mice, suggesting that Eomes^lo^ cells did not arise from Eomes^hi^ cells at day 10 (B16F10 vs. mock) (Figure 3D). This suggests that Eomes downregulation did not occur as a result of the transformation of Eomes^hi^ into Eomes^lo^ cells in the tumor microenvironment, which could indicate that Eomes^lo^ and Eomes^hi^ cells perhaps belonged to different lineages. Since ILC1s need T-bet for development and NK cells rely on it for maturation, we would anticipate the absence of ILC1s in T-bet knockout mice whereas immature NKs would be present. To test this hypothesis and to assess the source of Group 1 ILC subsets, we checked the profiles of Eomes^lo^ and Eomes^hi^ subsets in T-bet knockout mice. Since accumulation of these two subsets peaked at day 14 (Figure 2D), we compared cell numbers in T-bet knockout and wild type mice at this time point. Interestingly, we observed a significant reduction of Eomes^lo^ cell population in the lungs while Eomes^hi^ cells were the majority, supporting the hypothesis that Eomes^lo^ cells represent ILC1s while Eomes^hi^ cells are NK cells (Figure 3E).

### Murine Eomes^lo^ and Eomes^hi^ Group 1 ILC Subsets Share Phenotypic Similarities With ILC1s and cNKs, Respectively

**Figure 4:**
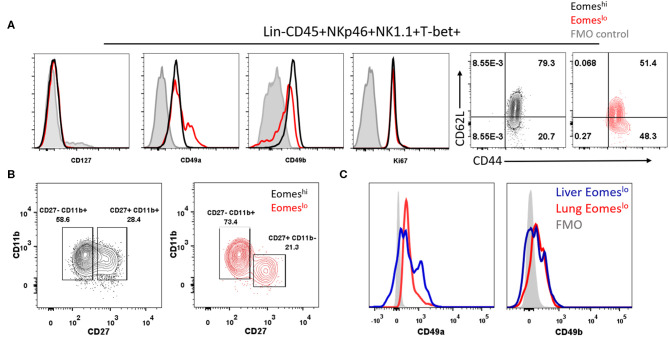
Eomes^lo^ Group 1 ILCs represent both cNK and tr-NK (ILC1) phenotype and function. **(A)** Representation of CD127, CD49a, CD49b expression in Eomes^lo^ and Eomes^hi^ subsets. Ki67 (proliferation marker) and CD44, CD62L (endothelial cell interaction/recruitment marker) levels on Eomes^lo^ and Eomes^hi^ cells were measured relative to FMO controls; red—Eomes^lo^, black—Eomes^hi^, gray—control. **(B)** Maturation status of NK cells using Cd11b and CD27 markers, with gating based on FMO controls; red—Eomes^lo^, black—Eomes^hi^ **(C)** CD49a and CD49b expression levels in lung Eomes^lo^ compared to liver Eomes^lo^ cells relative to FMO (Fluorescence Minus One) controls. MFI is Mean Fluorescence Intensity, *n* = 4 for each group. Data are representative of three independent repeats.

After establishing that Eomes^lo^ subset did not arise from Eomes^hi^ Group 1 ILCs, we sought to determine whether the T-bet^+^Eomes^hi^ and T-bet^+^Eomes^lo^ subsets represent conventional NK cells (cNK) and unconventional tissue resident NK cells (tr-NK)/ILC1, respectively, as in the liver and uterus (36, 54). To this end, we screened these subsets for various cell surface markers. Both of these subsets lacked the expression of CD127 (Figure 4A), a subunit of IL-7 receptor, suggesting that IL-7 was not needed for their maintenance. Next, we noted that Eomes^lo^ cells expressed higher levels of CD49a than Eomes^hi^ subset, pointing at an ILC1-like phenotype of Eomes^lo^ subset. Conversely, Eomes^hi^ cells expressed higher levels of CD49b than Eomes^lo^ subset suggesting an NK-like phenotype of Eomes^hi^ cells. Quantification of the percentage of CD49a^+^ and CD49b^+^ cells showed similar results (Figures S3A,B). We also measured cell proliferation and recruitment markers, Ki67 and CD44, CD62L, respectively (Figure 4A). We did not find any major difference in Eomes^lo^ and Eomes^hi^ subsets except that Eomes^hi^ cells comprised of 79.3% of double positive CD62L^+^CD44^+^ cells, suggesting their potential recruitment from other organs or the bloodstream while Eomes^lo^ cells consisted of 51.4% double positive population. Therefore, compared to Eomes^lo^ cells, the Eomes^hi^ subset showed larger proportion of cells with circulating cell phenotype. These observations are in alignment with an ILC1-like phenotype the Eomes^lo^ subset and NK-like phenotype for Eomes^hi^ cells as suggested by other studies (31). Next, in order to rule out the possibility that these Eomes^lo^ cells were immature NK cells, we examined the expression of NK maturation markers, CD11b and CD27, on these cells. While both Eomes^lo^ and Eomes^hi^ subsets consist of various fractions of immature NK cells (indicated by CD11b^−^CD27^+^ and CD11b^+^CD27^+^), the majority of Eomes^hi^ and Eomes^lo^ cells represented terminally mature cells (CD11b^+^CD27^−^) (Figure 4B), precluding the possibility that Eomes^lo^ subset cells were immature NK cells. Similar analysis of splenic Eomes^lo^ and Eomes^hi^ subsets showed a comparable profile for CD49b and CD49a (Figure S3C), whereas liver cell subsets showed higher expression of CD49a in liver Eomes^lo^ cells (Figure 4C).

### Eomes^lo^ Subset Has Reduced Effector Function Compared to Eomes^hi^ Group 1 ILCs

**Figure 5:**
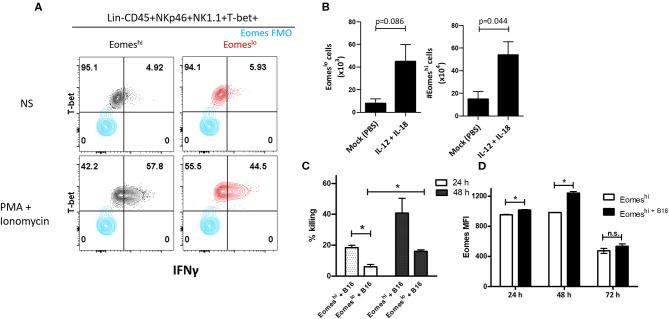
Eomes upregulation is correlated with Group 1 ILC-mediated cytotoxicity. **(A)** Representation of IFNγ expression in Eomes^lo^ and Eomes^hi^ subsets relative to FMO controls; red—Eomes^lo^, black—Eomes^hi^; Cells were isolated and stimulated *ex vivo* with PMA and Ionomycin (NS = non-stimulated) **(B)** Increase in Eomes^lo^ and Eomes^hi^ cell numbers in response to IL-12 and IL-18 stimulation *in vivo*. The lungs were harvested, and cells were counted at day 7. Here, *n* = 4 biological replicates. **(C)** Percentage killing of B16F10 melanoma cells by Eomes^hi^ subset relative to Eomes^lo^ cells at 24 and 48 h, normalized to spontaneous death (B16F10 only). **(D)** Measurement of Eomes MFI across 24, 48, and 72 h in flow-sorted murine Eomes^hi^ Group 1 ILC in co-culture with B16F10 cells compared to Eomes^hi^ subset alone. Cells were isolated from Eomes-GFP reporter mice; Eomes MFI is indicative of GFP MFI in the cells. Target to Effector ratio (T:E) was maintained at 4:1. MFI, Mean Fluorescence Intensity, *n* = 3 technical replicates, Data are representative of three independent repeats; data are presented as mean ± s.e.m.; Significance was tested using two-tailed students' *t*-test; n.s., not significant *p < 0.05.

**Figure 6:**
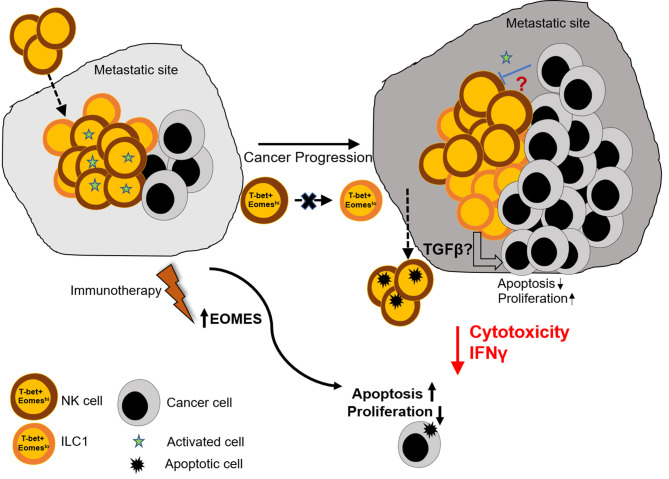
Graphical summary. Naïve lungs consist mainly of Eomes^hi^ NK cells. However, metastatic colonization leads to increase in the number of Eomes^lo^ ILC1. This increase in Eomes^lo^ ILC1s is associated with increased metastatic burden due to lower cytotoxic potential and reduced IFNγ production by this subset of ILC1s, compared to NK cells. This in turn supports proliferation of cancer cells and prevents apoptosis and cancer cell death. However, despite lowering of Eomes levels in Group 1 ILCs, Eomes^lo^ ILC1s did not seem to be derived from Eomes^hi^ NK cells, thus eliminating the possibility of plasticity under the conditions tested. Therefore, we propose that (i) the reduction in Eomes levels is associated with a worse metastatic burden and (ii) this could be due to production of immune-suppressive factors by Eomes^lo^ cells (iii) immunotherapeutic targets designed to augment Eomes levels could prove useful in the treatment of metastasized cancers.

In order to characterize the function of the two subsets, we measured IFNγ and TNFα produced by these cells. We did not observe measurable levels without cell stimulation, however, upon stimulation with PMA and Ionomycin, Eomes^lo^ cells produced significantly lower IFNγ compared to Eomes^hi^ cells, at day 14 (Figure 5A). Similarly, a larger fraction of Eomes^hi^ cells produced IFNγ compared to that of Eomes^lo^ cells (Figure S3D). On the other hand, TNFα did not show any difference in MFI (Figure S3E). This could be due to inherent differences in the nature of these cells or change in the activation status as a result of the polarization of the tumor microenvironment toward a pro-tumor milieu, albeit transient. Next, since Group 1 ILCs are activated by cytokines IL-12, IL-15, and IL-18 (8), we checked the response of NKp46^+^NK1.1^+^ subsets to these cytokines as per the treatment scheme (Figure S4A). Eomes^hi^ cells were more sensitive to the stimulation, and the cell numbers increased significantly compared to Eomes^lo^ cells upon stimulation with IL-12 + IL-18 (Figure 5B, Figure S4B). Since murine Eomes^lo^ ILC1-like subset produced lower IFNγ production compared to Eomes^hi^ subset, we next queried the role of Eomes in cytotoxicity of Group 1 ILCs. For this, we performed FACS sorting of Eomes^hi^ and Eomes^lo^ Group 1 ILCs and independently co-cultured them with B16F10 cells *ex vivo*. Interestingly, Eomes^hi^ cells were more cytotoxic compared to Eomes^lo^ at 24 and 48 h time points (Figure 5C). Furthermore, we observed an increase in the killing ability of Eomes^hi^ from 24 to 48 h with increase in intracellular Eomes expression (Figures 5C,D). This indicates that Eomes^lo^ ILC1s are less cytotoxic than Eomes^hi^ NK cells and cell cytotoxicity is positively associated with Eomes expression. Overall, we conclude that Eomes^lo^ and Eomes^hi^ Group 1 ILCs are fundamentally different and represent ILC1s and NK cells. Figure 6 illustrates our findings in a hypothetical model.

## Discussion

While Group 1 ILC subsets are considered to play an important role in cancer regulation due to their similarity to CD4 Th1 cells and production of IFNγ, their involvement and function in metastasis is rather unclear. This is due to phenotypic similarity to the well-known NK cells as well as evidence of plasticity among ILC subsets. In this study, we first identified ILC1 and NK cells in NSCLC patient blood using CD127 and CD56 markers. We further noted lower levels of Eomes in ILC1s compared to NK cells. Additionally, Eomes levels in NK cells were reduced with increase in disease severity. This prompted us to fully characterize Group 1 ILCs in murine models based on the expression of Eomes. Since Eomes and T-bet have been shown to be reliable in differentiating various subsets of Group 1 ILCs, we used these markers to study pulmonary Group 1 ILCs using a mouse model of B16F10 experimental metastasis. Since we observed a drop in Eomes levels in NK cells, post-metastasis (Stages III and IV), this murine model of metastasis was adopted to mirror similar conditions to confirm the role of Group 1 ILCs in cancer metastasis. Like in human cells, we noticed a decrease in the frequency of murine Eomes^hi^ NKp46^+^NK1.1^+^ cells with increase in tumor burden, giving rise to T-bet^+^Eomes^lo^ and T-bet^+^Eomes^hi^ ILC1 subsets in the lungs. Further phenotypic and functional characterization of these subsets revealed an ILC1-like signature for Eomes^lo^ subset and NK-like properties for Eomes^hi^ cells. Interestingly, none of the cellular subsets showed specificity for NK or ILC1 markers, thus resulting in an “intermediate” ILC1 population as has been reported recently (29). In alignment with this, adoptive transfer of Eomes^hi^ subset did not give rise to Eomes^lo^ subset, suggesting different lineage of the two cell types. Our findings are also in line with a study showing immune-evasion by cancer cells through conversion of NK cells into ILC1-like cells where Eomes^lo^ ILC1s produced significantly lower IFNγ and had reduced cytotoxicity compared to Eomes^hi^ NK cells (25). While we did not observe conversion of Eomes^hi^ cells into Eomes^lo^ cells under the conditions analyzed, the increase in the number of murine Eomes^lo^ ILC1s positively correlated with metastatic advancement. This observation, coupled with Eomes downregulation in human Group 1 ILCs with NSCLC progression, suggests that loss of Eomes is associated with a reduction in the anti-cancer effector function of Group 1 ILCs. Our findings provide an avenue for future elucidation of the molecular mechanism through which Eomes modulates cancer cell death. Additionally, it is interesting to note that the presence of B16F10 cells on the *in vivo* assays did not increase the levels of Eomes on ILC1 and NK cells (Figure 3C) but led to increased Eomes expression *ex vivo* in the killing assay (Figure 5D). We speculate that this perceived differences in the expression level could be due to different time points at which the cells were being analyzed. Eomes levels in Figure 3C were measured 6 days after adoptive transfer (at which point the Eomes levels could have stabilized) while in Figure 5D, the increase in eomes expression is detected at 24 and 48 h. It is also important to note that at 72 h time point in the killing assay, there was no observable change in Eomes levels, which further corroborates the *in vivo* data. Furthermore, while we observed near absence of Eomes^lo^ group 1 ILCs in T-bet KO mice, suggesting ILC1-like behavior, it is important to note that because of the compensatory nature of T-bet and Eomes, mice deficient in T-bet may have upregulated Eomes, which could in turn lead to their mis-identification as Eomes^hi^. Therefore, to ultimately define the developmental profile and origin of these cells, lineage tracing with knockout mice deficient in transcription factors crucial for development of ILC1 but not for NK cells must be carried out in future.

In the context of cancer, while it is conceivable that various group 1 ILC subsets play an anti-cancer effector function due to their IFNγ production, caution is needed since the modulation of the immune cell response might occur in the tumor microenvironment, thus changing the role of these cells from anti- to pro-tumor phenotype as reported for other immune cell types (55, 56). Furthermore, whether these murine Eomes^lo^ and Eomes^hi^ subsets are similar to human Eomes^lo^ ILC1s and Eomes^hi^ NK cells remains to be confirmed. While the phenotype and profile of various Group 1 ILC subsets (e.g., cNKs and ILC1), have been identified in the past decade in liver, thymus, kidney, uterus and skin, such information on lung Group 1 ILCs in metastasis is hitherto unavailable. Therefore, to our knowledge, this is the first study on the role of ILC1s in metastasis in human and mouse. Finally, although our studies do suggest that the phenotype of Eomes^lo^ and Eomes^hi^ cells resembles that of ILC1 and NK cells, respectively, we cannot preclude the possibility that these cells could be under different activation states of the same cell types which developed out of distinct lineages. Future studies involving determination of the lineage would help to further elucidate the development and function of these cells.

## Data Availability Statement

All datasets generated for this study are included in the article/Supplementary Material.

## Ethics Statement

The studies involving human participants were reviewed and approved by Domain Specific Review Board (DSRB Reference number 2016/00698) affiliated to the National Healthcare Group (NHG), Singapore. The patients/participants provided their written informed consent to participate in this study. Animal Studies were reviewed and approved by the Institutional Animal Care and Use Committee (IACUC), National University of Singapore, under Research Protocol R17-0209 and Breeding Protocol BR15-1142.

## Author Contributions

RV planned and performed the experiments. RV, JE, and RP analyzed the data. JS obtained patient consent and coordinated sample transfer between hospital and laboratory. RS, HM, and JT provided NSCLC samples and clinical information. RV, JE, and JD wrote the manuscript. All authors contributed to the article and approved the submitted version.

## Conflict of Interest

The authors declare that the research was conducted in the absence of any commercial or financial relationships that could be construed as a potential conflict of interest.

## References

[1] Takeda K Hayakawa Y Smyth MJ Kayagaki N Yamaguchi N Kakuta S. Involvement of tumor necrosis factor-related apoptosis-inducing ligand in surveillance of tumor metastasis by liver natural killer cells. Nat Med. (2001) 7:94–100. 10.1038/83416 11135622

[2] Walker J Barlow JL McKenzie ANJ. Innate lymphoid cells–how did we miss them? Nat Rev Immunol. (2013) 13:75–87. 10.1038/nri3349 23292121

[3] Spits H Artis D Colonna M Diefenbach A Di Santo JP Eberl G. Innate lymphoid cells–a proposal for uniform nomenclature. Nat Rev Immunol. (2013) 13:145–9. 10.1038/nri3365 23348417

[4] Schoenberger SP Katsikis PD Pulendran B. Crossroads Between Innate and Adaptive Immunity II: Preface. New York, NY: Springer-Verlag (2009).

[5] Hepworth MR. Innate lymphoid cell regulation: meeting the long-lost cousin. Trends Immunol. (2017) 38:873–4. 10.1016/j.it.2017.09.003 29029958

[6] Huang Q Seillet C Belz GT. Shaping innate lymphoid cell diversity. Front Immunol. (2017) 8:1569. 10.3389/fimmu.2017.01569 29201028 PMC5697340

[7] Nagasawa M Spits H Ros XR. Innate Lymphoid Cells. (ILCs): cytokine hubs regulating immunity and tissue homeostasis. Cold Spring Harb Perspect Biol. (2017) 10:a030304. 10.1101/cshperspect.a030304 29229782 PMC6280706

[8] Artis D Spits H. The biology of innate lymphoid cells. Nature. (2015) 517:293–301. 10.1038/nature14189 25592534

[9] Daussy C Faure F Mayol K Viel S Gasteiger G Charrier E. T-bet and Eomes instruct the development of two distinct natural killer cell lineages in the liver and in the bone marrow. J Exp Med. (2014) 211:563–77. 10.1084/jem.20131560 24516120 PMC3949572

[10] Townsend MJ Weinmann AS Matsuda JL Salomon R Farnham PJ Biron CA. T-bet regulates the terminal maturation and homeostasis of NK and Vα14i NKT cells. Immunity. (2004) 20:477–94. 10.1016/S1074-7613(04)00076-7 15084276

[11] Bernink JH Peters CP Munneke M te Velde AA Meijer SL Weijer K. Human type 1 innate lymphoid cells accumulate in inflamed mucosal tissues. Nat Immunol. (2013) 14:221–9. 10.1038/ni.2534 23334791

[12] Scoville SD Freud AG Caligiuri MA. Cellular pathways in the development of human and murine innate lymphoid cells. Curr Opin Immunol. (2019) 56:100–6. 10.1016/j.coi.2018.11.003 30579240 PMC7285385

[13] Sawa S Lochner M Satoh-Takayama N Dulauroy S Bérard M Kleinschek M. RORγt+ innate lymphoid cells regulate intestinal homeostasis by integrating negative signals from the symbiotic microbiota. Nat Immunol. (2011) 12:320–6. 10.1038/ni.2002 21336274

[14] Neumann K Karimi K Meiners J Voetlause R Steinmann S Dammermann W. A Proinflammatory role of type 2 innate lymphoid cells in murine immune-mediated hepatitis. J Immunol. (2016) 98:128–37. 10.4049/jimmunol.1600418 27872212

[15] Monticelli LA Artis D. Innate lymphoid cells promote lung tissue homeostasis following acute influenza virus infection. Nat Immunol. (2012) 12:1045–54. 10.1038/ni.2131 21946417 PMC3320042

[16] Nussbaum JC Van Dyken SJ von Moltke J Cheng LE Mohapatra A Molofsky AB. Type 2 innate lymphoid cells control eosinophil homeostasis. Nature. (2013) 502:245–8. 10.1038/nature12526 24037376 PMC3795960

[17] Salimi M Barlow JL Saunders SP Xue L Gutowska- Owsiak D Wang X. A role for IL-25 and IL-33-driven type-2 innate lymphoid cells in atopic dermatitis. J Exp Med. (2013) 210:2939–50. 10.1084/jem.20130351 24323357 PMC3865470

[18] Powell N Goldberg R Prescott N Lord GM Macdonald TT Powell N. The unusual suspects—innate lymphoid cells as novel therapeutic targets in IBD. Nat Publ Group. (2015) 12:271–83. 10.1038/nrgastro.2015.52 25971811

[19] Lund S Walford HH Doherty TA. Type 2 innate lymphoid cells in allergic disease. Curr Immunol Rev. (2013) 9:214–21. 10.2174/1573395510666140304235916 24876829 PMC4033554

[20] Mohapatra A Van Dyken SJ Schneider C Nussbaum JC Liang H-E Locksley RM. Group 2 innate lymphoid cells utilize the IRF4-IL-9 module to coordinate epithelial cell maintenance of lung homeostasis. Mucosal Immunol. (2016) 9:275–86. 10.1038/mi.2015.59 26129648 PMC4698110

[21] Leavy O. Innate-like lymphocytes: will the real ILC1 please stand up? Nat Rev Immunol. (2013) 13:67. 10.1038/nri3397 23348410

[22] Wills-Karp M Finkelman FD. Innate lymphoid cells wield a double-edged sword. Nat Immunol. (2011) 12:1025–7. 10.1038/ni.2142 22012433

[23] Spits H Bernink JH Lanier L. NK cells and type 1 innate lymphoid cells: partners in host defense. Nat Immunol. (2016) 17:758. 10.1038/ni.3482 27328005

[24] Klose CSN Flach M Möhle L Rogell L Hoyler T Ebert K. Differentiation of type 1 ILCs from a common progenitor to all helper-like innate lymphoid cell lineages. Cell. (2014) 157:340–56. 10.1016/j.cell.2014.03.030 24725403

[25] Gao Y Souza-Fonseca-Guimaraes F Bald T Ng SS Young A Ngiow SF. Tumor immunoevasion by the conversion of effector NK cells into type 1 innate lymphoid cells. Nat Immunol. (2017) 18:1004–15. 10.1038/ni.3800 28759001

[26] Dadi S Chhangawala S Whitlock BM Franklin RA Luo CT Oh SA. Cancer immunosurveillance by tissue-resident innate lymphoid cells and innate-like T cells. Cell. (2016) 164:365–77. 10.1016/j.cell.2016.01.002 26806130 PMC4733424

[27] Belz GT. ILC2s masquerade as ILC1s to drive chronic disease. Nat Publ Group. (2016) 17:611–12. 10.1038/ni.3467 27196511

[28] Bernink JH Krabbendam L Germar K de Jong E Gronke K Kofoed-Nielsen M. Interleukin-12 and−23 control plasticity Of Cd127+ group 1 and group 3 innate lymphoid cells in the intestinal lamina propria. Immunity. (2015) 43:146–60. 10.1016/j.immuni.2015.06.019 26187413

[29] Park E Patel S Wang Q Andhey P Zaitsev K Porter S. *Toxoplasma gondii* infection drives conversion of NK cells into ILC1-like cells. eLife. (2019) 8:e47605. 10.7554/eLife.47605 31393266 PMC6703900

[30] Cortez VS Cervantes-Barragan L Robinette ML Bando JK Wang Y Geiger TL. Transforming growth factor-β signaling guides the differentiation of innate lymphoid cells in salivary glands. Immunity. (2016) 44:1127–39. 10.1016/j.immuni.2016.03.007 27156386 PMC5114145

[31] Erick TK Brossay L. Phenotype and functions of conventional and non-conventional NK cells. Curr Opin Immunol. (2016) 38:67–74. 10.1016/j.coi.2015.11.007 26706497 PMC4715908

[32] Krneta T Gillgrass A Chew M Ashkar AA. The breast tumor microenvironment alters the phenotype and function of natural killer cells. Cell Mol Immunol. (2015) 13:1–12. 10.1038/cmi.2015.42 26277898 PMC5037278

[33] Geiger TL Abt MC Gasteiger G Firth MA O'Connor MH Geary CD. Nfil3 is crucial for development of innate lymphoid cells and host protection against intestinal pathogens. J Exp Med. (2014) 211:1723–31. 10.1084/jem.20140212 25113970 PMC4144732

[34] Mackay LK Minnich M Kragten NAM Liao Y Nota B Seillet C. Hobit and Blimp1 instruct a universal transcriptional program of tissue residency in lymphocytes. Science. (2016) 352:459–63. 10.1126/science.aad2035 27102484

[35] Weizman O-E Adams NM Schuster I Krishna C Pritykin Y Lau C. ILC1 confer early host protection at initial sites of viral infection. Cell. (2017) 171:795–808.e12. 10.1016/j.cell.2017.09.052 29056343 PMC5687850

[36] Gordon SM Chaix J Rupp LJ Wu J Madera S Sun JC. The transcription factors T-bet and eomes control key checkpoints of natural killer cell maturation. Immunity. (2012) 36:55–67. 10.1016/j.immuni.2011.11.016 22261438 PMC3381976

[37] Lazarevic V Glimcher LH Lord GM. T-bet: a bridge between innate and adaptive immunity. Nat Rev Immunol. (2013) 13:777–89. 10.1038/nri3536 24113868 PMC6290922

[38] Vivier E Artis D Colonna M Diefenbach A Di Santo JP Eberl G. Innate lymphoid cells: 10 years on. Cell. (2018) 174:1054–66. 10.1016/j.cell.2018.07.017 30142344

[39] O'Sullivan TE. Dazed and confused: NK cells. Front Immunol. (2019) 10:2235. 10.3389/fimmu.2019.02235 31616419 PMC6763593

[40] Silver JS Kearley J Copenhaver AM Sanden C Mori M Yu L Inflammatory triggers associated with exacerbations of COPD orchestrate plasticity of group 2 innate lymphoid cells in the lungs. Nat Immunol. (2016) 17:626–35. 10.1038/ni.3443 27111143 PMC5345745

[41] Boulenouar S Michelet X Duquette D Brenner MB Andrian U Von Boulenouar S. Adipose type one innate lymphoid cells regulate macrophage homeostasis through targeted article adipose type one innate lymphoid cells regulate macrophage homeostasis through targeted cytotoxicity. Immunity. (2017) 46:273–86. 10.1016/j.immuni.2017.01.008 28228283

[42] O'Sullivan TE Rapp M Fan X Weizman O-E Bhardwaj P Adams NM. Adipose-resident group 1 innate lymphoid cells promote obesity-associated insulin resistance. Immunity. (2016) 45:1–14. 10.1016/j.immuni.2016.06.016 27496734 PMC5004886

[43] Crome SQ Nguyen LT Lopez-Verges S Yang SYC Martin B Yam JY. A distinct innate lymphoid cell population regulates tumor-associated T cells. Nat Med. (2017) 23:368–75. 10.1038/nm.4278 28165478 PMC5497996

[44] López-Soto A Gonzalez S Smyth MJ Galluzzi L. Control of metastasis by NK cells. Cancer Cell. (2017) 32:135–54. 10.1016/j.ccell.2017.06.009 28810142

[45] Nicholson SE Keating N Belz GT. Natural killer cells and anti-tumor immunity. Mol Immunol. (2017) 110:40–47. 10.1016/j.molimm.2017.12.002 29233542

[46] Vacca P Munari E Tumino N Moretta F Pietra G Vitale M Human natural killer cells and other innate lymphoid cells in cancer: friends or foes? Immunol Lett. (2018) 201:14–19. 10.1016/j.imlet.2018.11.004 30439479

[47] Carrega P Campana S Bonaccorsi I Ferlazzo G. The Yin and Yang of innate lymphoid cells in cancer. Immunol Lett. (2016) 179:29–35. 10.1016/j.imlet.2016.06.003 27296768

[48] van Beek J Martens A Bakdash G de Vries I. Innate lymphoid cells in tumor immunity. Biomedicines. (2016) 4:7. 10.3390/biomedicines4010007 28536374 PMC5344245

[49] Loyon R Jary M Salomé B Gomez-Cadena A Galaine J Kroemer M. Peripheral innate lymphoid cells are increased in first line metastatic colorectal carcinoma patients: a negative correlation with Th1 immune responses. Front Immunol. (2019) 10:2121. 10.3389/fimmu.2019.02121 31555301 PMC6742701

[50] Zhang J Marotel M Fauteux-Daniel S Mathieu A-L Viel S Marçais A. T-bet and Eomes govern differentiation and function of mouse and human NK cells and ILC1. Eur J Immunol. (2018) 48:738–50. 10.1002/eji.201747299 29424438

[51] Collins A Rothman N Liu K Reiner SL. Eomesodermin and T-bet mark developmentally distinct human natural killer cells. JCI Insight. (2017) 2:e90063. 10.1172/jci.insight.90063 28289707 PMC5333970

[52] Huang R-L Teo Z Chong HC Zhu P Tan MJ Tan CK. ANGPTL4 modulates vascular junction integrity by integrin signaling and disruption of intercellular VE-cadherin and claudin-5 clusters. Blood. (2011) 118:3990–4002. 10.1182/blood-2011-01-328716 21841165

[53] Flavell RA Sanjabi S Wrzesinski SH Licona-Limón P. The polarization of immune cells in the tumour environment by TGFβ. Nat Rev Immunol. (2010) 10:554–67. 10.1038/nri2808 20616810 PMC3885992

[54] Sojka DK Plougastel-Douglas B Yang L Pak-Wittel MA Artyomov MN Ivanova Y. Tissue-resident natural killer (NK) cells are cell lineages distinct from thymic and conventional splenic NK cells. eLife. (2014) 3:e01659. 10.7554/eLife.01659 24714492 PMC3975579

[55] Johansson M DeNardo DG Coussens LM. Polarized immune responses differentially regulate cancer development. Immunol Rev. (2008) 222:145–54. 10.1111/j.1600-065X.2008.00600.x 18363999 PMC2494984

[56] Hobson-Gutierrez SA Carmona-Fontaine C. The metabolic axis of macrophage and immune cell polarization. Dis Model Mech. (2018) 11:dmm034462. 10.1242/dmm.034462 29991530 PMC6124558

