# Supplementary material for: Eomes Expression Defines Group 1 Innate Lymphoid Cells During Metastasis in Human and Mouse
Source: Front Immunol. 2020 Jun 17;11:1190. doi: 10.3389/fimmu.2020.01190 (PMC7311635; doi:10.3389/fimmu.2020.01190)
Supplement: Supplementary file 1 [file Data_Sheet_1.docx]

**Supplementary data (Figures S1, S2, S3, S4 and Table S1) manuscript ID: 537767**

**Eomes expression defines Group 1 Innate Lymphoid Cells during metastasis in human and mouse**

**Figure S1**

**
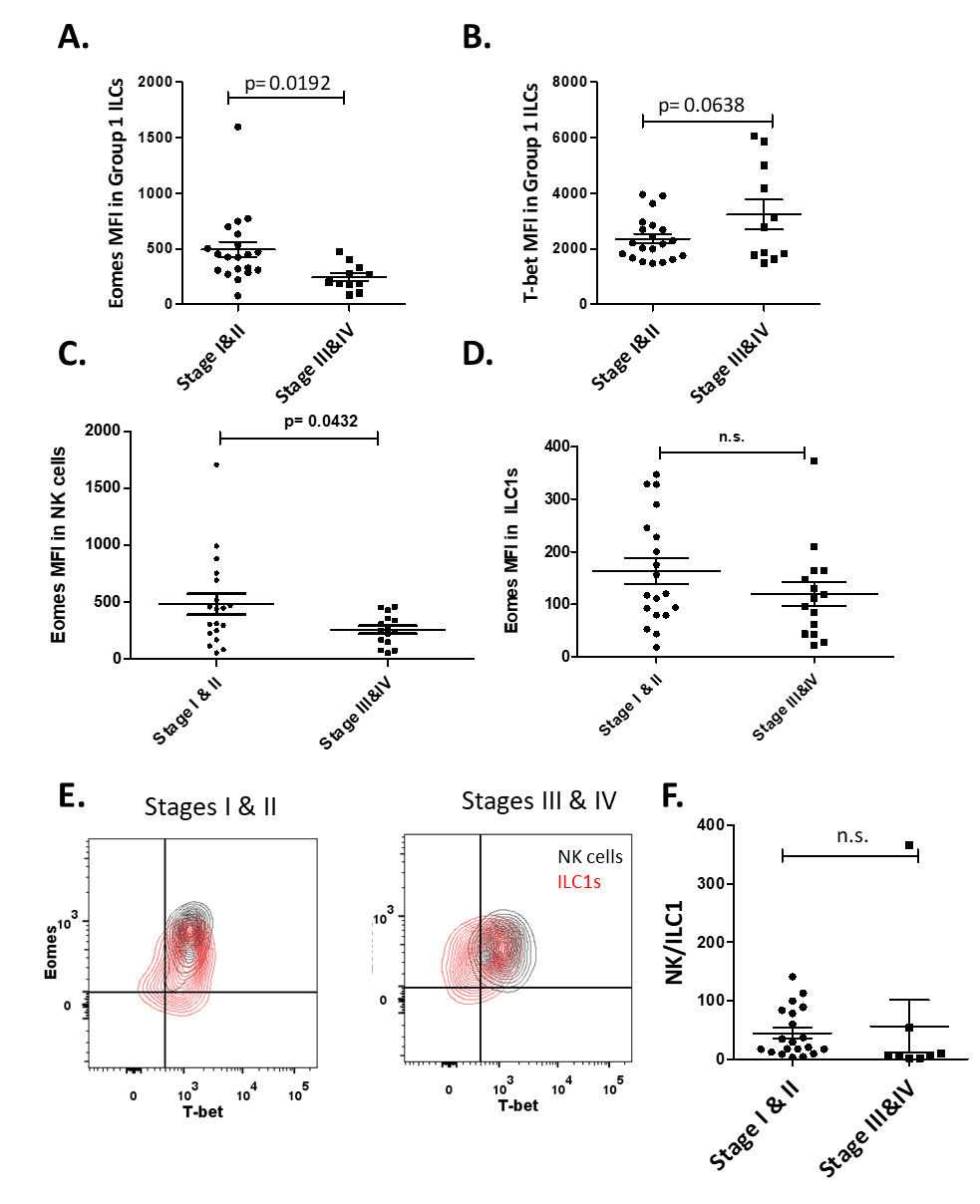
**

**Figure S1. Profiling of human Group 1 ILCs during NSCLC progression. A.** Eomes and **B.** T-bet MFI in CD45^+^ Lineage (CD3, CD19, CD11b, CD11c) ^-^ c-Kit^-^ CRTH2^-^ Group 1 ILCs in pre-metastatic (Stage I and II) and post-metastasis (Stage III and IV). **C.** Eomes MFI in CD127^-^CD56^+^ NK cells and **D.** Eomes MFI in CD127^+^CD56^-^ ILC1s gated over CD45^+^ Lineage (CD3, CD19, CD11b, CD11c) ^-^ c-Kit^-^ CRTH2^-^ Group 1 ILCs. ILC1s. **E.** Flow plot representation of Eomes^lo^ ILC1 and Eomes^hi^ NK cells and **F.** Ratio of CD127^-^CD56^+^ NK/ CD127^+^CD56^-^ ILC1 during early stages I & II and late stages, III & IV. Here, n= 16 for stage I, n=4 for stage II, n=4 for stage III, n=7 for stage IV. Data are presented as mean ± s.e.m.; significance was tested using two tailed students’ t-test.

**Figure S2**


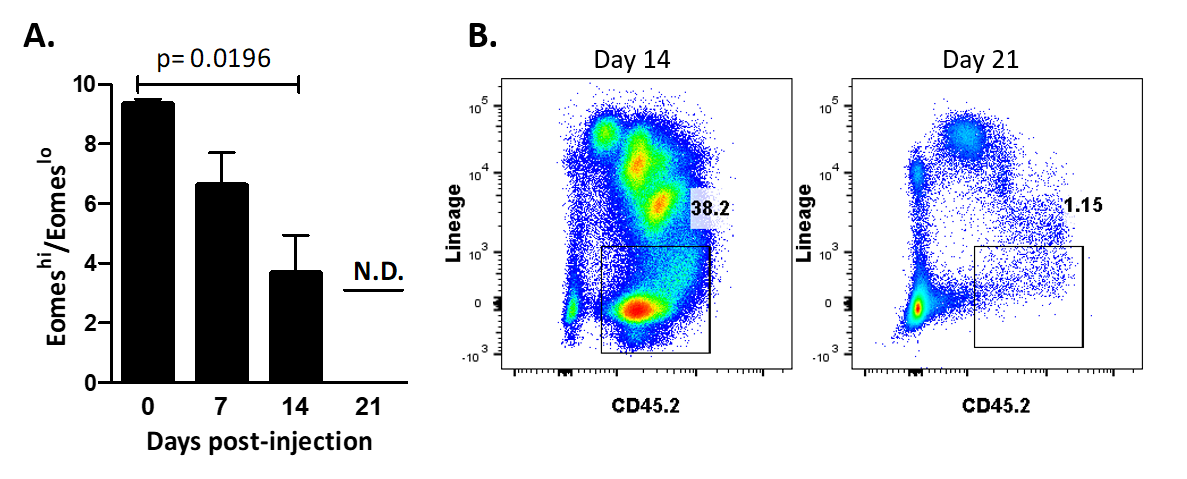


**Figure S2. Eomes^lo^ Group 1 ILCs accumulate in the lung during B16F10 metastatic progression A.** Ratio of Eomes^hi^ to Eomes^lo^ cells at different time points post injection. Due to increase in number of Eomes^lo^ cells, the ratio of the two subsets decreases with increase in metastatic burden. **B.** Reduction in CD45.2^+^ cells at day 21 compared to day 14 post injection of B16F10 cells. Due to reduced cell number, we could not detect sufficient numbers of Eomes^hi^/Eomes^lo^ cells at day 21. Here, n=4 for each group. Data is representative of 3 independent repeats; Data are presented as mean ± s.e.m.; statistical significance was tested using two-tailed students’ t-test.

**Figure S3**


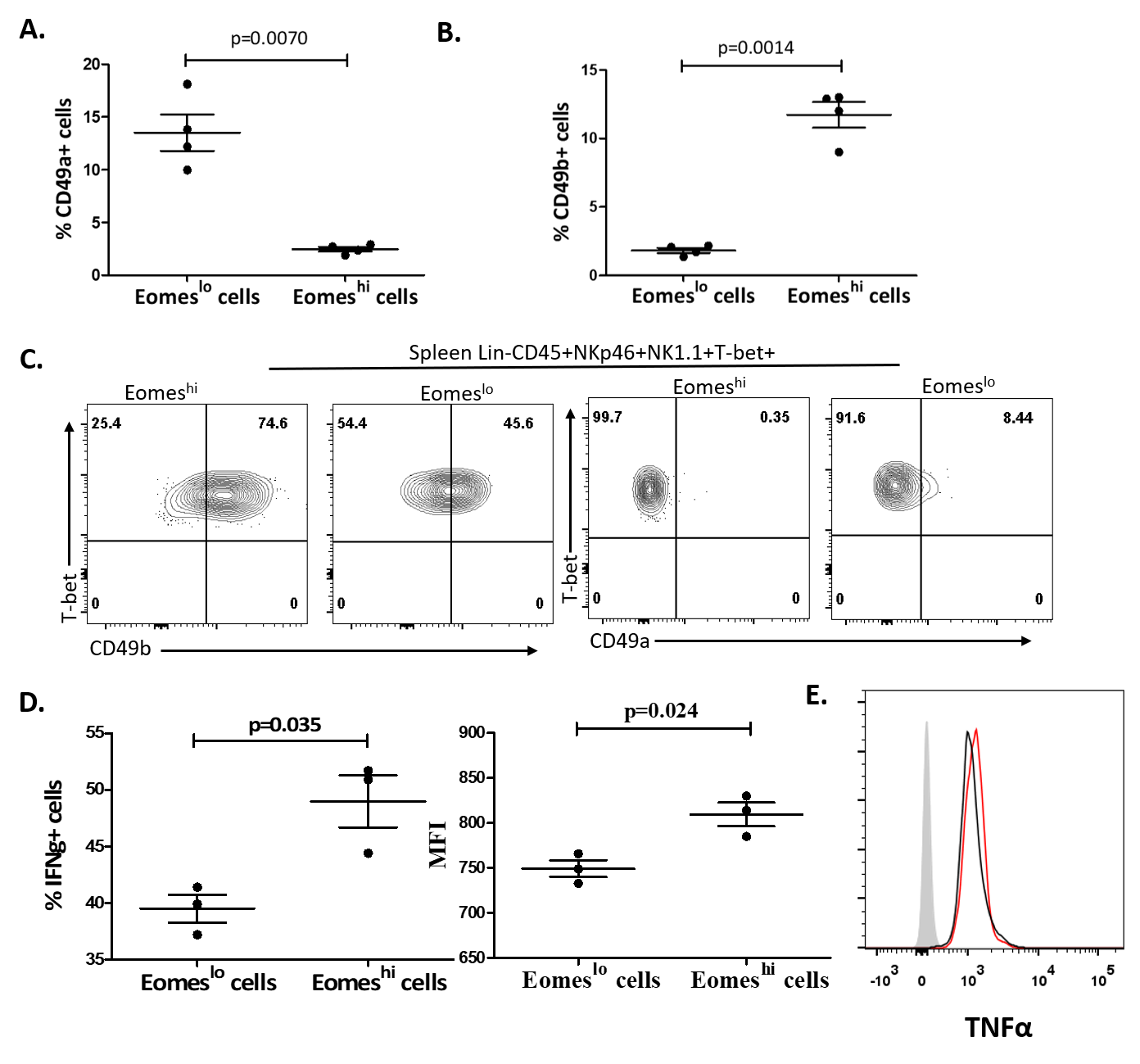


**Figure S3. Eomes^lo^ Group 1 ILCs represent both cNK and tr-NK (ILC1) phenotype and function. A.** Quantification of CD49a^+^ cells in pulmonary Eomes^lo^ and Eomes^hi^ subsets at day 14 post B16F10 injections; Eomes^lo^ subsets has a larger fraction of CD49a^+^ cells suggesting their ILC1-like phenotype. **B.** Quantification of CD49b^+^ cells in pulmonary Eomes^lo^ and Eomes^hi^ subsets at day 14 post B16F10 injections; Eomes^hi^ subsets has a larger fraction of CD49b^+^ cells suggesting their NK-like phenotype. **C.** Representation of CD49b and CD49a expression in splenic Eomes^lo^ and Eomes^hi^ subsets. **D.** Quantification of IFNγ producing cells and MFI in lung Eomes^lo^ and Eomes^hi^ subsets. **E.** Histogram showing TNFα MFI in lung Eomes^lo^ and Eomes^hi^ subsets. Here, red – Eomes^lo^, black – Eomes^hi^, grey – FMO. Cells were isolated and stimulated *ex vivo* with PMA and Ionomycin. MFI is Mean Fluorescence Intensity, n=3 for each group. Data is representative of 3 independent repeats; Data are presented as mean ± s.e.m.; statistical significance was tested using two-tailed students’ t-test.

**Figure S4**


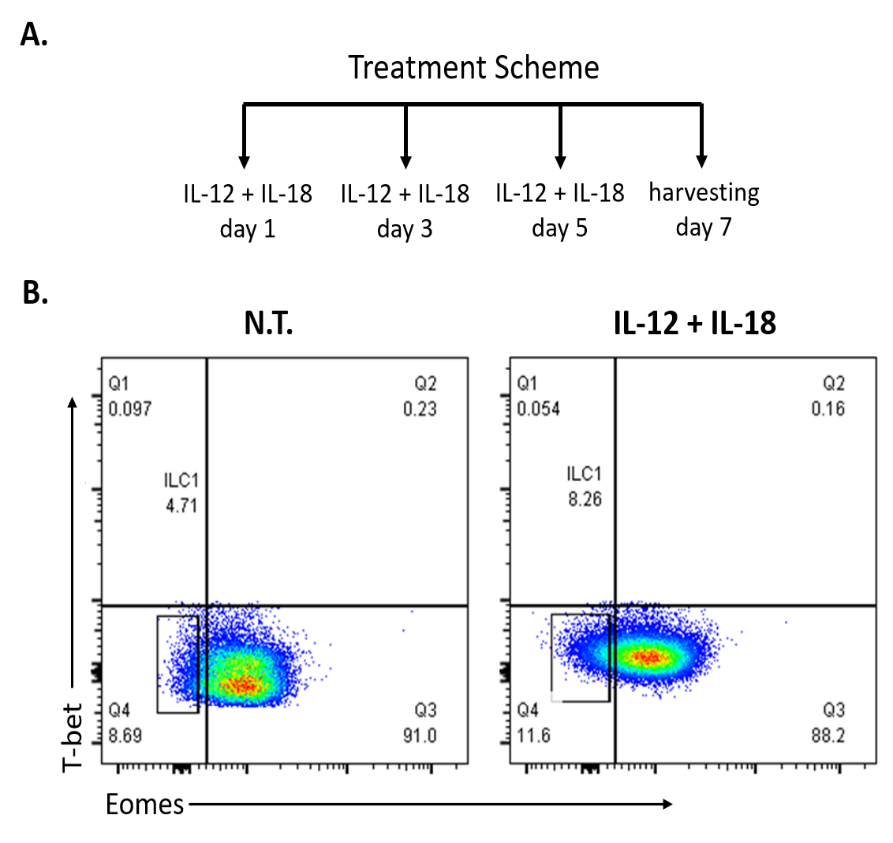


**Figure S4. Treatment scheme and gating for ex vivo cytokine stimulation**

**A.** Mice were given 50 μl of 0.5 μg IL-12 and 1.0 μg IL-18 in 1x PBS intranasally on days 1, 3 and 5, followed by harvesting of lung tissues on day 7. **B.** Flow plot representation of increase in Eomes^lo^ and Eomes^hi^ cell numbers in response to IL-12 and IL-18 stimulation *in vivo*.

**Table S1: Cancer staging and Patient information**
